# *Mycobacterium lepromatosis* Lepromatous Leprosy in US Citizen Who Traveled to Disease-Endemic Areas

**DOI:** 10.3201/eid2405.171972

**Published:** 2018-05

**Authors:** Ajay Vir Singh, Devendra Singh Chauhan

**Affiliations:** ICMR-National JALMA Institute for Leprosy and Other Mycobacterial Diseases, Agra, India

**Keywords:** *Mycobacterium lepromatosis*, Lepromatous, leprosy, bacteria, United States

**To the Editor:** The article by Virk et al. (*1*) highlighted that a person can acquire *Mycobacterium lepromatosis* infection without exposure to a person infected with leprosy or to known vectors during short stays (2 trips of 7 days each over 3 calendar years) in Mexico. The authors then concluded that *M. lepromatosis* lepromatous leprosy is a travel-related hazard for travelers to Mexico or other disease-endemic areas. We note that the exact source of acquiring the *M. lepromatosis* infection by the patient in this study was entirely uncertain, and experimental evidence was not enough to prove *M. lepromatosis* to be a travel-related hazard.

In contrast, Jessamine et al. (*2*) reported *M. lepromatosis* infection and leprosy-like illness in a patient in Canada who had no history of contact or travel to leprosy-endemic areas. Jessamine et al. indicated that transmission dynamics of *M. lepromatosis* infection is complex, and undiscovered mechanisms or unknown reservoir interactions may exist in such areas of nonendemic regions. Previous studies have also reported the roles of subclinical cases and environmental reservoirs in the transmission of leprosy (*3*,*4*). However, Virk et al. have not disentangled other possible sources (existence of unrecognized subclinical cases, contact with hidden leprosy cases, and environmental reservoir) of prolonged exposure of *M. lepromatosis* to the study patient in his vicinity. Thus, the assertion of Virk et al. (*1*) that United States citizens can acquire *M. lepromatosis* when traveling to Mexico or other leprosy-endemic areas as tourists is misleading and demands extensive research to prove it.

In addition, it is intriguing to note that host genetic determinants can influence the acquisition and onset of leprosy (*5*). Therefore, the inference of a single case study cannot be generalized for all citizens of the United States. The data from these reports suggest that the epidemiologic studies of leprosy in nonendemic areas should consider travel history to delineate this issue.

## References

[1] Virk A, Pritt B, Patel R, Uhl JR, Bezalel SA, Gibson LE, et al. *Mycobacterium lepromatosis* lepromatous leprosy in US citizen who traveled to disease-endemic areas. Emerg Infect Dis. 2017;23:1864–6. 10.3201/eid2311.171104 29048278 PMC5652441

[2] Jessamine PG, Desjardins M, Gillis T, Scollard D, Jamieson F, Broukhanski G, et al. Leprosy-like illness in a patient with *Mycobacterium lepromatosis* from Ontario, Canada. J Drugs Dermatol. 2012;11:229–33.https://www.ncbi.nlm.nih.gov/entrez/query.fcgi?cmd=Retrieve&db=PubMed&list_uids=22270208&dopt=Abstract 22270208

[3] Araújo S, Lobato J, Reis EM, Souza DO, Gonçalves MA, Costa AV, et al. Unveiling healthy carriers and subclinical infections among household contacts of leprosy patients who play potential roles in the disease chain of transmission. Mem Inst Oswaldo Cruz. 2012;107(Suppl 1):55–9. 10.1590/S0074-02762012000900010 23283454

[4] Mohanty PS, Naaz F, Katara D, Misba L, Kumar D, Dwivedi DK, et al. Viability of *Mycobacterium leprae* in the environment and its role in leprosy dissemination. Indian J Dermatol Venereol Leprol. 2016;82:23–7. 10.4103/0378-6323.168935 26728806

[5] Alter A, Alcaïs A, Abel L, Schurr E. Leprosy as a genetic model for susceptibility to common infectious diseases. Hum Genet. 2008;123:227–35. 10.1007/s00439-008-0474-z 18247059

